# Fetal exposure to maternal inflammation interrupts murine intestinal development and increases susceptibility to neonatal intestinal injury

**DOI:** 10.1242/dmm.040808

**Published:** 2019-10-01

**Authors:** Timothy G. Elgin, Erin M. Fricke, Huiyu Gong, Jeffrey Reese, David A. Mills, Karen M. Kalantera, Mark A. Underwood, Steven J. McElroy

**Affiliations:** 1 Department of Pediatrics, University of Iowa, Iowa City, IA 52242, USA; 2 Department of Obstetrics and Gynecology, University of Iowa, Iowa City, IA 52242, USA; 3 Department of Pediatrics, Vanderbilt University, Nashville, TN 37232, USA; 4 Department of Food Science and Technology, University of California Davis, Davis, CA 95616, USA; 5 Department of Pediatrics, University of California Davis, Sacramento, CA 95817, USA; 6 Department of Microbiology and Immunology, University of Iowa, Iowa City, IA 52242, USA

**Keywords:** Chorioamnionitis, LPS, Goblet, Paneth, IL-6

## Abstract

Fetal exposure to chorioamnionitis can impact the outcomes of the developing fetus both at the time of birth and in the subsequent neonatal period. Infants exposed to chorioamnionitis have a higher incidence of gastrointestinal (GI) pathology, including necrotizing enterocolitis (NEC); however, the mechanism remains undefined. To simulate the fetal exposure to maternal inflammation (FEMI) induced by chorioamnionitis, pregnant mice (C57BL/6J, *IL-6*^−/−^, *RAG*^−/−^ or *TNFR1*^−/−^) were injected intraperitoneally on embryonic day (E)15.5 with lipopolysaccharide (LPS; 100 µg/kg body weight). Pups were delivered at term, and reared to postnatal day (P)0, P7, P14, P28 or P56. Serum and intestinal tissue samples were collected to quantify growth, inflammatory markers, histological intestinal injury, and goblet and Paneth cells. To determine whether FEMI increased subsequent susceptibility to intestinal injury, a secondary dose of LPS (100 µg/kg body weight) was given on P5, prior to tissue harvesting on P7. FEMI had no effect on growth of the offspring or their small intestine. FEMI significantly decreased both goblet and Paneth cell numbers while simultaneously increasing serum levels of IL-1β, IL-10, KC/GRO (CXCL1 and CXCL2), TNF and IL-6. These alterations were IL-6 dependent and, importantly, increased susceptibility to LPS-induced intestinal injury later in life. Our data show that FEMI impairs normal intestinal development by decreasing components of innate immunity and simultaneously increasing markers of inflammation. These changes increase susceptibility to intestinal injury later in life and provide novel mechanistic data to potentially explain why preterm infants exposed to chorioamnionitis prior to birth have a higher incidence of NEC and other GI disorders.

## Abstract (precis)

**Summary:** Fetal exposure to maternal inflammation alters normal development of neonatal goblet and Paneth cells, and significantly increases intestinal injury in the neonatal period through IL-6-dependent pathways.

## INTRODUCTION

Human chorionic membranes play an important role in providing protection, nutrition and endocrine support to the developing fetus (Moore et al., 2006; Kim et al., 2007; Goldstein et al., 2017). If these membranes become compromised, the consequences can be severe and far reaching for the developing infant. Intrauterine infection is associated with up to 40% of premature births (Agrawal and Hirsch, 2012), as well as pathology in several fetal tissues, including the cerebral white matter (Wu et al., 2003), adrenal gland (Gover et al., 2013), eyes (Woo et al., 2012), lungs (Lee et al., 2015; Hudalla et al., 2018), heart (Park et al., 2015), kidneys (Hudalla et al., 2018) and immune system (Weitkamp et al., 2016; Hudalla et al., 2018). Of particular interest is the association between exposure to intrauterine infection and the later development of necrotizing enterocolitis (NEC). Multiple studies have suggested that exposure to chorioamnionitis is a risk factor for the development of NEC (Been et al., 2013; García-Muñoz Rodrigo et al., 2014), and we have recently shown that exposure of the fetus to lipopolysaccharide (LPS)-induced maternal inflammation results in fetal intestinal injury (Fricke et al., 2018). However, the mechanisms linking inflammation of the fetal membranes and NEC remain undefined.

NEC is a devastating disease of prematurity. In the United States, NEC affects more than 4000 premature infants each year, has a mortality rate of nearly 30% and an associated annual cost of over US$1-billion (McElroy, 2014). A leading hypothesis of NEC pathogenesis involves a combination of immune dysregulation, intestinal inflammation and bacterial translocation (Vongbhavit and Underwood, 2016). Chorioamnionitis may contribute to the development of NEC through activation of the maternal innate immune system, which provokes a strong downstream fetal inflammatory response, creating a proinflammatory environment in the neonate which may then compromise normal neonatal developmental patterns (Gantert et al., 2010; Yamada et al., 2015; Hudalla et al., 2018).

To further explore the relationship between maternal inflammation and subsequent gastrointestinal (GI) injury in her offspring, we injected pregnant mice with *E. coli*-derived LPS to simulate the inflammatory environment seen in chorioamnionitis (Fricke et al., 2018). Maternal LPS exposure is a well-established model of both chorioamnionitis and preterm labor (Fricke et al., 2018; McCarthy et al., 2018), and has been shown to induce the fetal immune response and increase cytokine levels in fetal samples (Kuypers et al., 2012; Fricke et al., 2018; Hudalla et al., 2018). Our model has been shown to induce increases in placental and serum cytokines, placental injury, and fetal inflammation that model what is seen in human chorioamnionitis. Further, the fetal effects of this model are not caused by direct LPS exposure as we found no evidence that LPS was able to cross the placenta (Fricke et al., 2018). While there is some controversy in the field as to whether LPS can cross the placenta and how to best model chorioamnionitis in a rodent, our objective was to understand the effects of fetal exposure to maternal inflammation (FEMI) on the long-term development of the neonatal intestinal tract. Thus, we chose this model as its dose-dependent LPS-mediated induction of maternal inflammation is a good proxy for the inflammatory surge seen during chorioamnionitis without having to control for off-target bacterial effects. Using this model, we aimed to explore the relationship between FEMI and subsequent intestinal injury through evaluation of intestinal development, systemic markers of inflammation, and susceptibility to intestinal injury in the offspring neonates. We hypothesized that FEMI causes initial injury to the fetal intestine, inducing a pro-inflammatory alteration of the developmental program of the neonatal small-intestinal tract, and that FEMI would result in an increased susceptibility to neonatal inflammation (NI)-induced intestinal injury. Our data show that FEMI alters the normal developmental pattern of neonatal goblet and Paneth cells, two intestinal epithelial cells that are key to innate immunity. We also found that FEMI significantly increases susceptibility and severity of intestinal injury in the neonatal period, and that these effects are ameliorated in animals that lack the ability to signal through IL-6 pathways. These data support novel mechanistic hypotheses to potentially explain why preterm infants exposed to chorioamnionitis prior to birth have a higher incidence of NEC and other GI disorders.

## RESULTS

### FEMI has minimal impact on growth or intestinal measurements

**Fig. 1.**
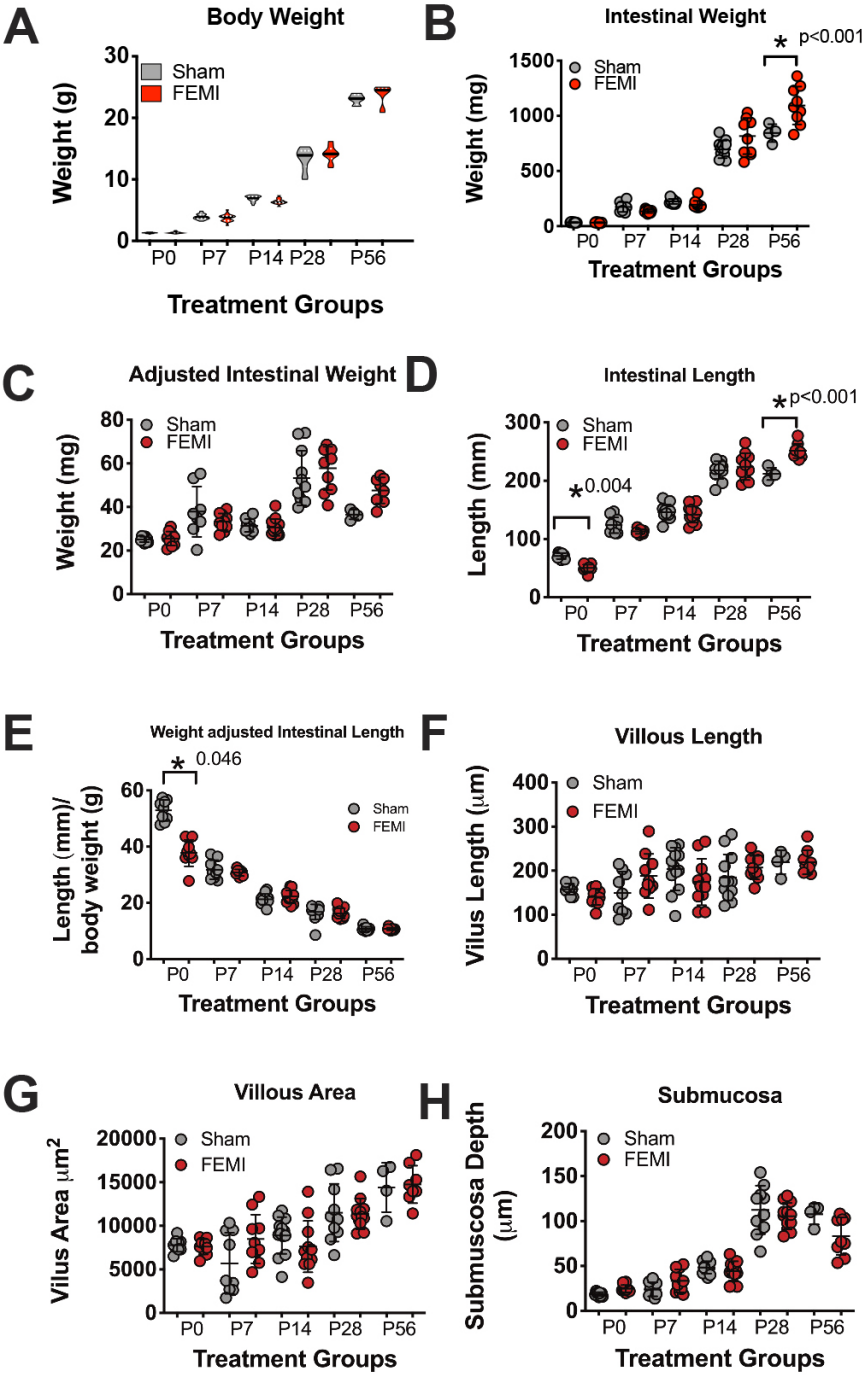
**Fetal exposure to maternal inflammation has marginal effects on growth and on small-intestinal histology.** The body weight of pups exposed to FEMI were no different than controls. (A) Violin plot of sham and FEMI-exposed animals at each age. *N* for each group measured was P0=57, P7=41, P14=45, P28=22, P56=13. Small-intestinal samples harvested from sham and FEMI-exposed animals were quantified for (B) intestinal weight, (C) intestinal weight adjusted for body weight, (D) intestinal length, (E) intestinal length adjusted for body weight, (F) average villous length, (G) average villous area and (H) average submucosal depth. Sample numbers for each age group were P0=19, P7=18, P14=23, P28=22, P56=13. All histologic measurements represented counting 300 crypts per animal. FEMI exposure induced significant increases in intestinal weight at P28 and P56 (B), but this effect was lost when correcting for body weight (C). A significant decrease in intestinal length was seen at P0 in FEMI-exposed animals compared to sham controls (*P*=0.046), and a significant increase in intestinal length was seen in FEMI-exposed animals compared to sham controls at P56 (*P*=0.0002). However, the difference at P56 was lost when adjusted for body weight (E). No significant differences were observed in histologic measurements (F-H).

To assess whether fetal exposure to LPS-induced maternal inflammation (FEMI) would result in altered growth dynamics, pups were weighed weekly from birth until day of life 56 (P56). As male C57BL/6J mice become significantly larger than females around 4 weeks of age (data from https://www.jax.org/jax-mice-and-services/strain-data-sheet-pages/body-weight-chart-000664), we only included male animals at P28 and P56 time points. Weights were not significantly different between FEMI and sham groups at any age (Fig. 1A). We next assessed the effect of FEMI on the structure of the small intestine itself. FEMI mouse small-intestinal samples were significantly heavier at P56 (*P*<0.001) than control samples; however, when intestinal weights were adjusted for body weight, this significance disappeared (Fig. 1B,C). FEMI had significant effects on small-intestinal length at P0 (sham 70.9 mm versus FEMI exposed 50.3 mm, *n*=10 for each group, *P*=0.04) and P56 (sham 211.8 mm versus FEMI exposed 251.3 mm, *n*=4 for sham and *n*=9 for FEMI, *P*=<0.001), but all other ages were similar to controls (Fig. 1D). When adjusted for body weight, FEMI-induced effects on intestinal length remained at P0, but disappeared at P56 (Fig. 1E). Although FEMI impacted neonatal growth, it had no effect on the size of the small-intestinal microarchitecture as there were no differences seen in villous length, villous area or submucosal depth (Fig. 1F-H).

### FEMI significantly decreases quantities of goblet and Paneth cells

**Fig. 2.**
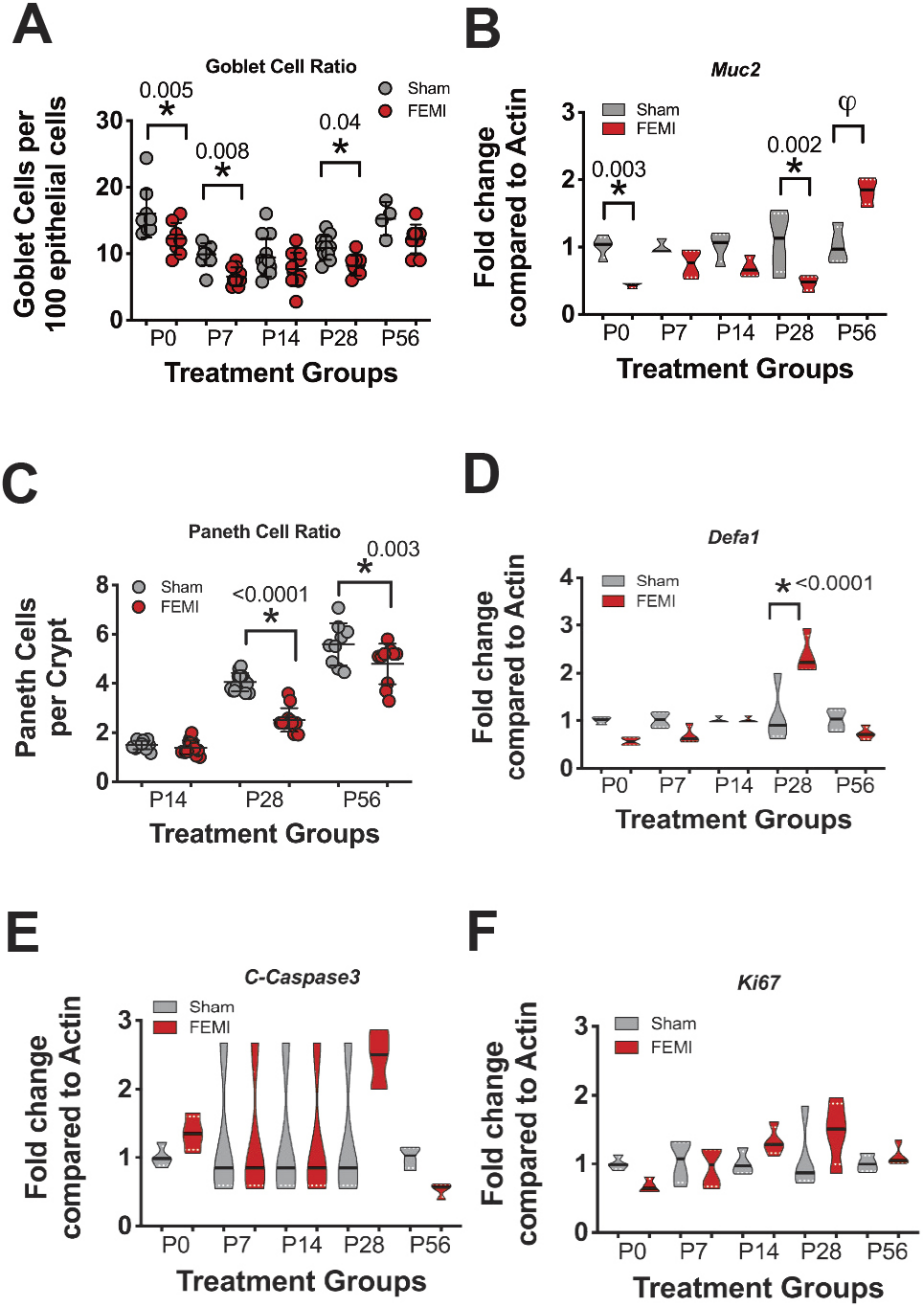
**FEMI exposure results in decreased goblet and Paneth cell populations later in life.** (A) Harvested intestinal tissue samples were quantified for goblet cell populations as a ratio of enterocytes. FEMI exposure caused significantly decreased goblet cell ratios compared to sham controls at P0 (FEMI 12% versus sham 16%, *P*=0.005, *n*=17), at P7 (FEMI 6.6% versus sham 9.9%, *P*=0.008, *n*=20) and at P28 (FEMI 8.2% versus sham 11%, *P*=0.04, *n*=25). (B) Gene expression of the goblet cell marker *Muc2* was quantified (*n*=8 at each time point). FEMI significantly decreased *Muc2* expression at P0 and P28 (*P*=0.003 and 0.002 respectively). *Muc2* expression was also significantly increased at P56, but the increase was less than a twofold change and was thus considered clinically non-significant (φ). (C) Tissues were also quantified for Paneth cell populations per intestinal crypt. FEMI exposure significantly decreased Paneth cell numbers at P28 (2.5 versus 4.1, *P*<0.0001) and at P56 (4.8 versus 5.6, *P*=0.003). (D) Gene expression of the Paneth cell marker *Defa1* was quantified (*n*=8 at each time point). *Defa1* expression was significantly elevated at P28 (*P*<0.0001). Gene expression for (E) cleaved caspase 3 and (F) *Ki67* were also quantified to examine potential effects on apoptosis and proliferation (*n*=8 at each time point). All time points were similar between sham and FEMI-exposed animals.

We next quantified the number of goblet and Paneth cells to determine whether FEMI would affect secretory intestinal epithelial cells that are important to barrier defense of the small intestine. FEMI significantly decreased mucus-positive goblet cell populations (Fig. 2A) at P0, P7 and P28 (*P*=0.007, 0.01 and 0.01, respectively). Quantification of *Muc2*, which codes for mucin 2, the predominant mucus-producing gene in the small intestine, showed that FEMI significantly decreased *Muc2* expression at P0 (*P*=0.03) and at P28 (*P*=0.002) (Fig. 2B). Paneth cells, which appear in C57BL/6 mice after P10, were also significantly reduced at P28 and P56 (*P*≤0.0001 and 0.003, respectively) following FEMI exposure (Fig. 2C). FEMI also significantly increased *Defa1* expression at P28 (*P*<0.0001) (Fig. 2D). *Defa1* codes for α-1 defensin, one of the antimicrobial peptides produced by Paneth cells. FEMI induced no changes at any time in cleaved caspase-3, an important regulator of apoptosis, or *Ki67*, an important regulator of cellular proliferation (Fig. 2E,F).

### FEMI induces an increase in neonatal baseline serum markers of inflammation

**Fig. 3.**
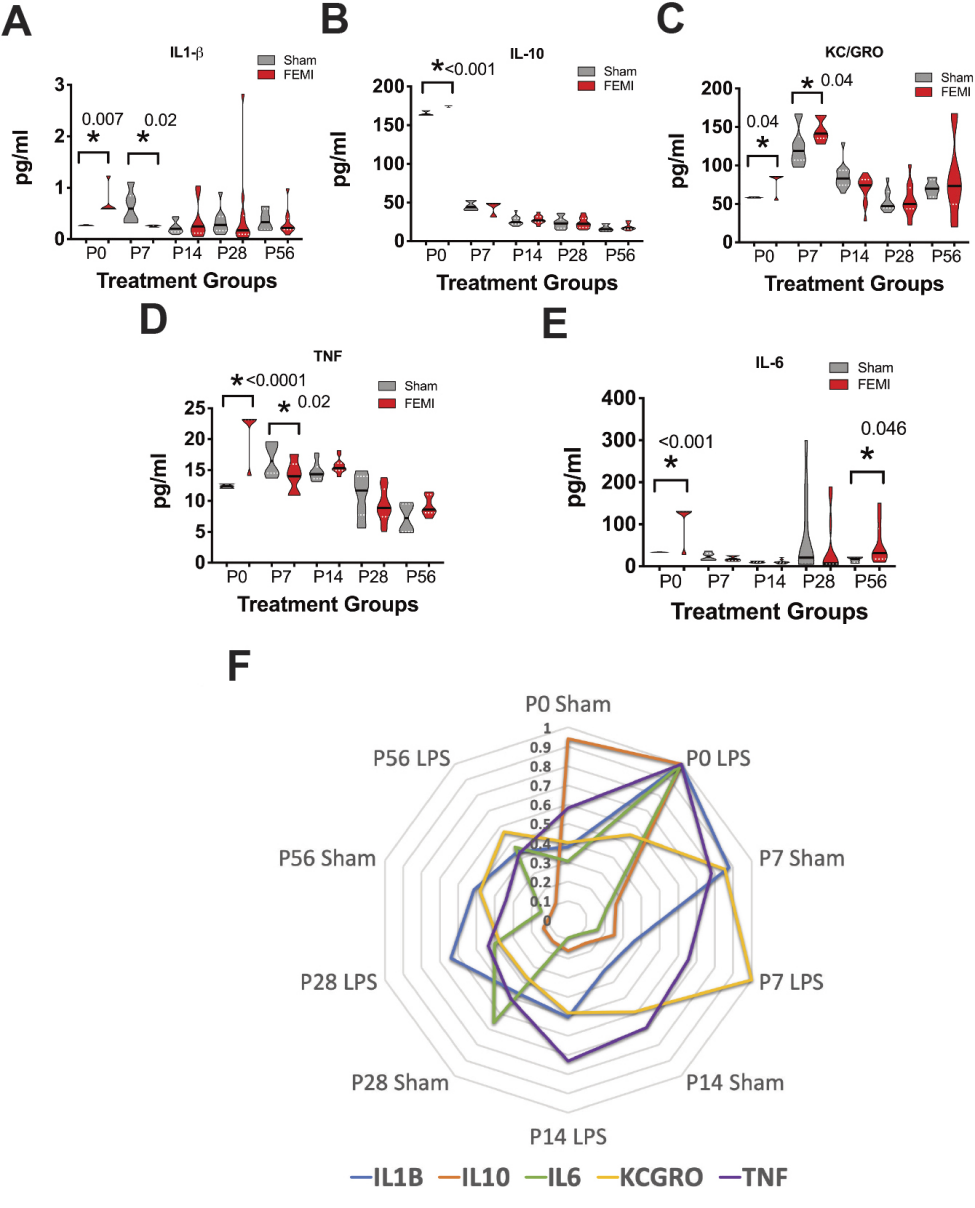
**FEMI results in significant acute and chronic elevation in cytokines.** Serum samples were obtained from animals exposed to FEMI and compared to sham at the ages shown. Serum was quantified for (A) IL-1β, (B) IL-10, (C) KC/GRO, (D) TNF and (E) IL-6. *N* for all cytokines measured was P0=19, P7=20, P14=26, P28=22, P56=14. FEMI induced significant increases in all cytokines compared to sham controls at P0. Interestingly, FEMI exposure also resulted in significantly decreased IL-1β (*P*=0.02), significantly decreased TNF (*P*=0.02) and significantly increased KC/GRO (*P*=0.04) at P7. Lastly, FEMI induced a significant increase in IL-6 at P56 (*P*=0.046). (F) Data from A-E represented as a radar plot by age to show general trends relative to each other. All cytokines are plotted as % of maximal value.

Our prior studies have shown that FEMI induces significant small-intestinal injury at and after birth (Fricke et al., 2018). To assess whether FEMI also results in increased markers of inflammation, serum inflammatory cytokines [IL-6, TNF, KC/GRO (CXCL1 and CXCL2), IL-10 and Il-1β] were measured at all time points (*n*=10-13 for all treatment groups) (Fig. 3). Significant increases were seen at P0 in all cytokines measured in FEMI animals compared to controls (IL-1β *P*=0.007, IL-10 *P*<0.001, KC/GRO *P*=0.4, TNF *P*<0.0001 and IL-6 *P*<0.001). However, later ages were cytokine dependent. At P7, IL-1β and TNF were both significantly decreased compared to sham controls (*P*=0.02 and 0.02, respectively), while KC/GRO levels were still significantly elevated compared to sham controls (*P*=0.04). However, the most interesting may be IL-6, which had similar values from P7-P28 but, by P56, despite no secondary intervention, had significantly elevated IL-6 levels in FEMI animals compared to controls (FEMI 51.8 pg/ml versus sham 16.1 pg/ml, *P*=0.046, *n*=10 FEMI and 4 sham).

### Effects of FEMI on the offspring are independent of microbiome alterations

**Fig. 4.**
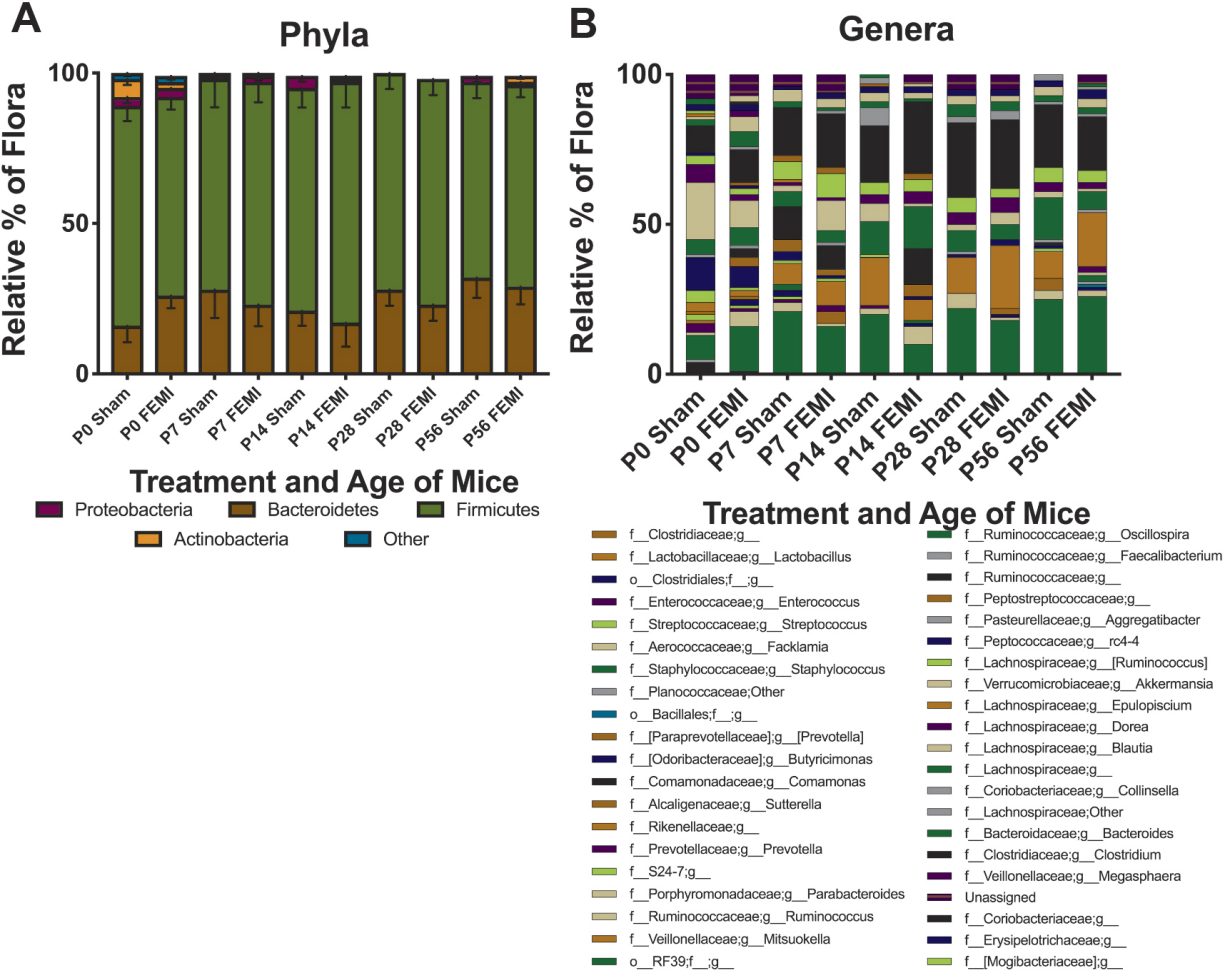
**FEMI has no significant effects on the long-term composition of the cecal microbiome.** Cecal samples were obtained from animals exposed to FEMI and sham at time points shown (*n*=10-14 per time point). Samples were sequenced and analyzed for composition at the (A) phyla and (B) genera levels. No statistical differences between FEMI-exposed and sham controls were seen at any age group. o, order; f, family; g, genus.

To determine whether FEMI induces offspring effects through alterations in the intestinal microbiome, cecal samples were analyzed for microbial composition at the phyla and genera levels, and were compared to sham control animals. Neither the phyla level nor the genera level showed any significant differences in microbial composition following fetal exposure to maternal inflammation compared to sham animals (Fig. 4).

### FEMI induces increased susceptibility to subsequent LPS-induced neonatal intestinal injury

**Fig. 5.**
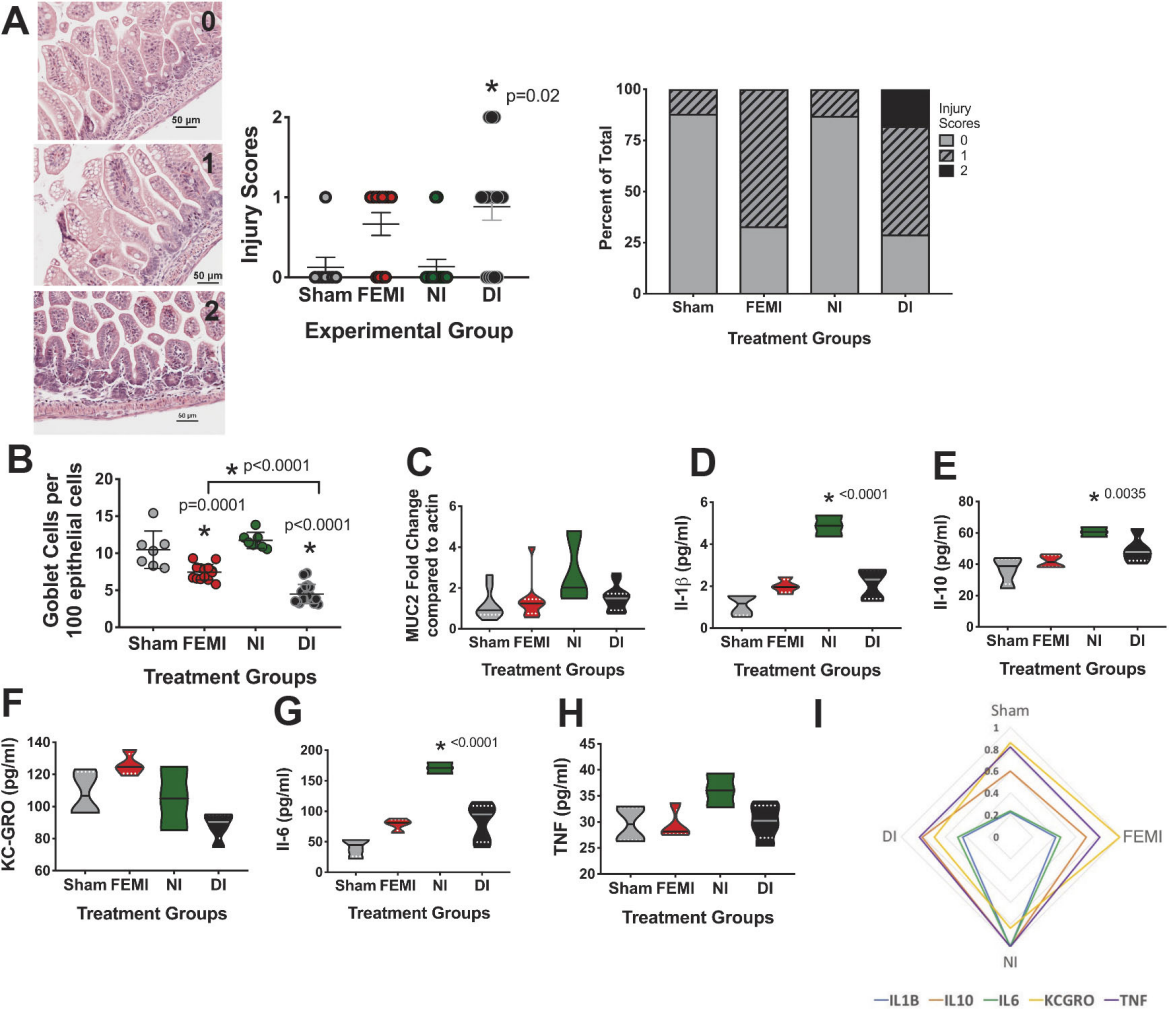
**FEMI induces an increased susceptibility to subsequent LPS-induced intestinal injury.** C57BL/6J mice were exposed to 100 µg/kg body weight LPS as a fetus (FEMI; E15.5), as a neonate (NI; P5), or both (DI) and compared to sham controls. *N* for all experiments was sham=7, NI=7, FEMI=14, DI=17. All animals were raised until P7. (A) Animals exposed to DI were the only animals to have intestinal injury that was significantly greater than controls (*P*=0.02). The left panel shows example histological scores (0, 1 and 2). The center panel shows individual animal injury scores while the right panel shows the percentage at which each level of injury occurred. (B) Animals exposed to either FEMI (*P*=0.0001) or DI (*P*<0.0001) showed significantly decreased mucus-positive goblet cells per 100 intestinal epithelial cells, with DI-exposed animals having significantly fewer goblet cells than animals treated with FEMI alone (*P*<0.0001). (C) However, no significant differences were seen in *Muc2* expression levels. Serum samples were quantified for (D) IL-1β, (E) IL-10, (F) KC/GRO, (G) IL-6 and (H) TNF. NI exposure induced significant elevations of IL-1β (*P*<0.0001), IL-10 (*P*=0.0035) and IL-6 (*P*<0.0001). No other treatments induced significant changes. (I) Data from D-H represented as a radar plot by age to show general trends relative to each other. All cytokines are plotted as % of maximal value.

Since FEMI induces baseline inflammation and intestinal injury in newborn animals, we next evaluated the response to a direct secondary exposure to LPS-induced inflammation. Pregnant dams were exposed to LPS or an equivalent volume of saline at E15.5 as above. Following birth, offspring were randomized to be given an additional injection with either LPS (100 µg/kg body weight) or an equal volume of saline on P5. No mice experienced mortality from LPS exposure at P5. This created four experimental groups: sham controls (sham; maternal saline:neonatal saline), FEMI (maternal LPS:neonatal saline), NI (maternal saline:neonatal LPS) and double inflammation (DI; maternal LPS:neonatal LPS). Following the second injection, animals were returned to their mother for 48 h prior to euthanasia and tissue harvesting. Animals exposed to DI demonstrated significant elevation in injury scores compared with sham (*P*=0.0217, *n*=17) (Fig. 5A). FEMI (*P*=0.0001) and DI (*P*<0.0001) animals also had significantly fewer mucus-positive goblet cells than sham controls, whereas those exposed to only NI did not (Fig. 5B). Although mucus-positive goblet cell numbers were altered, *Muc2* expression was not significantly different given any of the treatment groups (Fig. 5C).

We next evaluated the effects of fetal (FEMI), neonatal (NI) and combined (DI) inflammatory exposure on serum markers of inflammation (Fig. 5D-I). When measured on P7, exposure to NI significantly increases serum levels of IL-1β, IL-10 and IL-6 (*P*<0.0001, 0.0035 and <0.0001, respectively) when compared to sham controls. While DI exposure caused a modest elevation in levels of IL-1β, IL-10 and IL-6 compared to sham controls, these did not reach significance.

### FEMI-induced intestinal injury and loss of goblet cells at P7 are IL-6 dependent

**Fig. 6.**
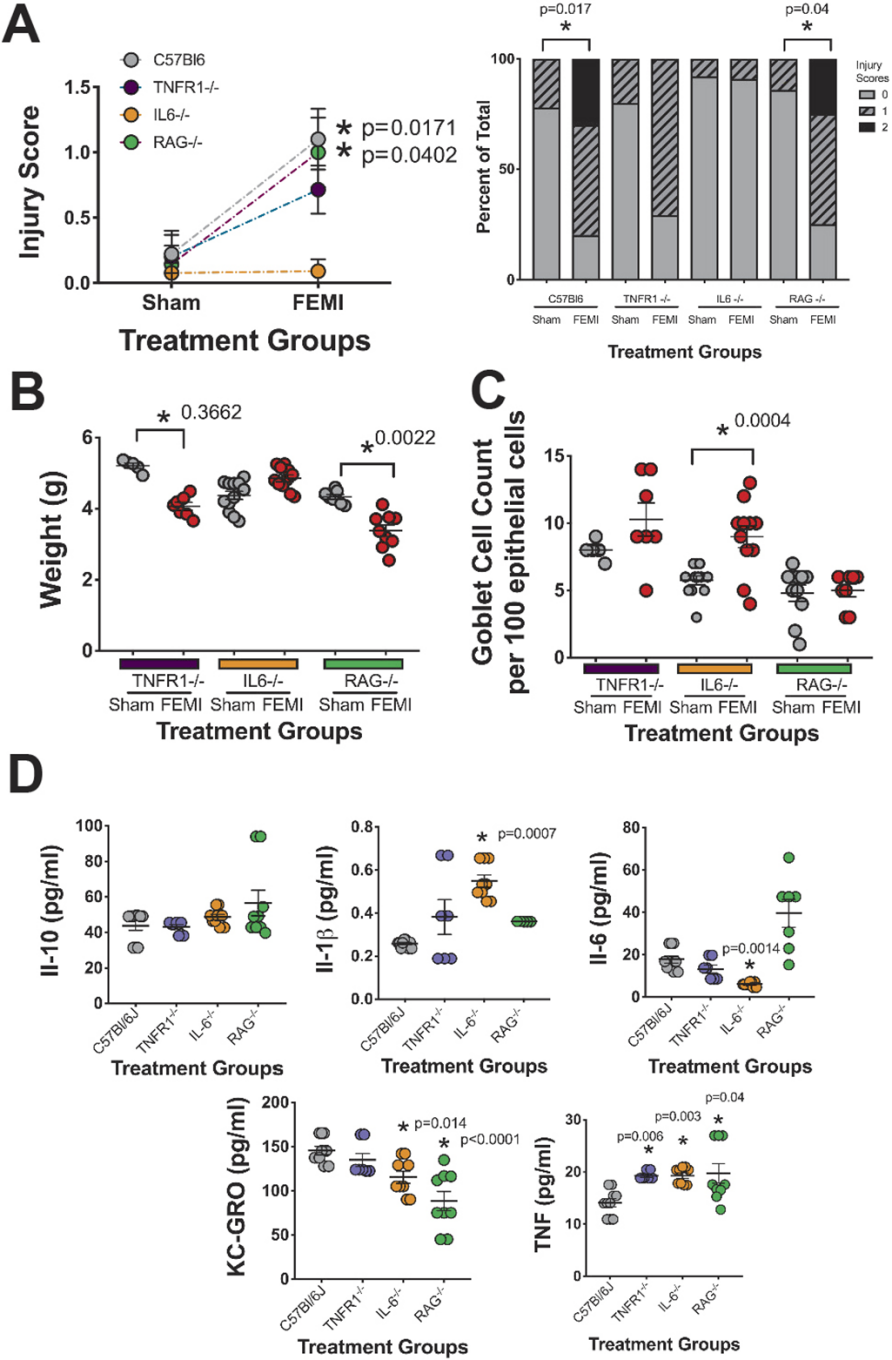
**FEMI-induced intestinal injury and loss of goblet cells at P7 are IL-6 dependent.** Wild-type C57BL/6J, *TNFR1*^−/−^, *IL-6*^−/−^ and *RAG*^−/−^ pregnant mice were injected with 100 µg/kg body weight LPS at E15.5, and their pups were delivered normally and raised without intervention until P7. *N* for all experiments were wild type=20, *TNFR1*^−/−^=12, *IL-6*^−/−^=23 and *RAG*^−/−^=18. (A) FEMI induced significant intestinal injury at P7 only in C57BL/6J wild-type (*P*=0.0171) and *RAG*^−/−^ (*P*=0.0402) mice. *TNFR1*^−/−^ mice showed a non-significant trend in increased injury, while *IL-6*^−/−^ mice had injury scores equivalent to sham controls. The left panel shows individual animal injury scores while the right panel shows the percentage values of each level of injury. (B) While wild-type animals exhibited no change in weight (Fig. 1), both *TNFR1*^−/−^ and *RAG*^−/−^ animals exhibited significant weight loss following exposure to FEMI (*P*=0.3662 and 0.0022, respectively). (C) While FEMI wild-type C57BL/6J animals had significantly decreased numbers of goblet cells at P7 (Fig. 3), *TNFR1*^−/−^ and *RAG*^−/−^ mice showed no difference. In contrast, FEMI *IL-6*^−/−^ mice experienced a significant increase in goblet cell numbers (*P*=0.0004). (D) Serum samples were quantified for IL-1β, IL-10, KC/GRO, IL-6 and TNF. FEMI wild-type C57BL/6J animals were compared statistically to FEMI knockout animals. FEMI *TNFR1*^−/−^ animals had significantly higher levels of serum TNF (*P*=0.006). FEMI *IL-6*^−/−^ animals had significantly higher levels of serum IL-1β (*P*=0.0007) and TNF (*P*=0.003), and significantly lower serum levels of IL-6 (*P*=0.0014) and KC/GRO (*P*=0.014). FEMI *RAG*^−/−^ mice had significantly lower levels of serum KC/GRO (*P*<0.0001) and higher levels of serum TNF (*P*=0.04).

We and others have previously shown that LPS can induce maternal inflammation but does not cross the placenta to directly impact the fetus (Wolfs et al., 2014; Fricke et al., 2018; Hudalla et al., 2018). Thus, we next wanted to examine whether one or more of the maternal cytokines generated by LPS-induced inflammation was causing the intestinal effects seen in our model. To examine this, wild-type C57BL/6J, TNF receptor 1 (*TNFR1*)^−/−^, *IL-6*^−/−^ and *RAG*^−/−^ pregnant mice (all on C57BL/6J backgrounds) were injected with LPS as above and their pups were allowed to deliver normally. Pups were raised without intervention until P7 when all pups were euthanized. At P7, we examined growth and intestinal injury, and determined small-intestinal goblet cell numbers. *IL-6*^−/−^ mice were protected from FEMI-induced intestinal injury and had injury scores equivalent to sham control animals. *RAG*^−/−^ mice caused similar intestinal injury as in wild-type C57BL/6J mice, while *TNFR1*^−/−^ animals had a phenotype that was in between (Fig. 6A). Our prior data from Fig. 1 had shown that C57BL/6J animals exposed to FEMI gained more weight over time than sham but, at P7, were equivalent. At P7, *IL-6*^−/−^ animals exposed to FEMI had no significant differences in weight compared to shams, but both *TNFR1*^−/−^ and *RAG*^−/−^ FEMI animals experienced significant weight loss compared to sham controls (Fig. 6B). We had previously seen that wild-type C57BL/6J animals exposed to FEMI had significantly decreased numbers of goblet cells at P7 (Fig. 2). FEMI *RAG*^−/−^ mice showed no difference at P7, while FEMI *IL-6*^−/−^ mice experienced a significant increase in goblet cell numbers and FEMI *TNFR1*^−/−^ showed a trend toward an increase compared to sham controls (Fig. 6C). FEMI *TNFR1*^−/−^ animals had a significant increase in serum TNF levels (*P*=0.006) compared to FEMI C57BL/6J animals, while FEMI *IL-6*^−/−^ animals had significant increases in IL-1β (*P*=0.0007) and TNF (*P*=0.003) along with significantly less IL-6 (*P*=0.0014) compared to FEMI C57BL/6J controls (Fig. 6D). Lastly, FEMI *RAG*^−/−^ animals experienced greater overall variability than other animals and had significantly less KC/GRO (*P*<0.0001) and significantly higher TNF (*P*=0.04) than FEMI C57BL/6J controls.

## DISCUSSION

Chorioamnionitis remains a serious problem as it is associated with up to 40% of premature births (Agrawal and Hirsch, 2012). Fetal exposure to chorioamnionitis is associated with increased risk of pathology in multiple organ systems of the offspring, including the intestine (Wu et al., 2003; Woo et al., 2012; Gover et al., 2013; Lee et al., 2015; Weitkamp et al., 2016; Hudalla et al., 2018). This is of special importance in the preterm infant as it has also been associated with increased development of NEC (Been et al., 2013; García-Muñoz Rodrigo et al., 2014). However, mechanisms linking inflammation of the fetal membranes and NEC remain undefined. NEC's pathophysiology remains incompletely defined but is thought to involve a combination of immune dysregulation, intestinal inflammation and the alteration of the fetal microbiome (Vongbhavit and Underwood, 2016). Our hypothesis was that FEMI, which simulates chorioamnionitis (Fricke et al., 2018), would cause initial injury to the fetal intestine, causing a pro-inflammatory alteration to the normal developmental program of intestinal defense systems such as goblet cells, Paneth cells, intestinal microbial composition and control of intestinal inflammatory regulation. Our data show that FEMI does indeed impair the normal developmental pattern of goblet and Paneth cells, two intestinal epithelial cells that are key to innate immunity. We also found that the effects of FEMI are IL-6 dependent, and that FEMI significantly increases susceptibility and severity of subsequent intestinal injury. These data provide novel mechanistic data to potentially explain why preterm infants exposed to chorioamnionitis prior to birth have a higher incidence of NEC and other GI disorders.

Our data show that exposure to FEMI induces a complex inflammatory response in the fetus. While FEMI induces fetal intestinal injury (Fricke et al., 2018) as well as increases in multiple markers of inflammation, including serum levels of IL-1β, TNF, KC/GRO (the mouse homolog of IL-8) and IL-6, it simultaneously reduces the number of mucin-positive goblet cells and the expression of *Muc2*, which codes for the most prominent mucin produced in the small intestine. Intestinal mucins are large glycoproteins that act as an important factor in protection of the small intestine from bacterial invasion. Mucins provide a physical barrier, facilitate bacterial removal and help to concentrate digestive-aiding enzymes (Hecht, 1999). While the role mucins play in the pathogenesis of NEC remains incompletely elucidated, decreases in goblet cells have been seen in infants who have developed NEC (McElroy et al., 2011) and in rodent models of NEC (Clark et al., 2005; Zhang et al., 2012). Of greater concern is that FEMI mice not only have a decreased number of goblet cells at birth, but throughout much of the neonatal period. While FEMI did not induce alterations in the composition of the microbiome, loss of the protective mucin barrier will render the host more susceptible to inflammation and tissue invasion by intraluminal microbes.

This defensive deficit is compounded by a concomitant decrease in normal development of Paneth cells and the expression of *Defa1*, a representative gene coding for antimicrobial α-defensins. Like goblet cells, Paneth cells are an integral component to the health and homeostasis of the small intestine (Bevins and Salzman, 2011; Clevers and Bevins, 2013). Paneth cells contribute to homeostasis in several ways, including: maintaining a semi-sterile intestinal crypt niche; regulating mucosal development; bolstering the host defense; and shaping the intestinal microbiome (Salzman et al., 2007; Bevins and Salzman, 2011; Clevers and Bevins, 2013; Bel et al., 2017). Impairment of the function of these cells has significant adverse consequences, including a reduction in clearance of bacterial pathogens (Sherman et al., 2005; Vaishnava et al., 2008) and the development of inflammatory bowel disease (Bel et al., 2017; Delorme-Axford and Klionsky, 2018). Thus, any delay or disruption of Paneth cell development is likely to impair the ability of the intestine to prevent bacteria from moving into and infecting tissues that are normally sterile. Our data show that FEMI induces a decreased number of Paneth cells up to 6 weeks after birth. The loss of both Paneth and goblet cells may help to explain the finding of elevations in baseline serum cytokines during normal development (Fig. 4). As our lab has shown that Paneth cell disruption in mice followed by intestinal exposure to *Klebsiella pneumonia* can induce NEC-like pathology (McElroy et al., 2014; White et al., 2017b; Lueschow et al., 2018), and our lab and others have shown that a loss of Paneth cells is associated with the diagnosis of human NEC (Coutinho et al., 1998; McElroy et al., 2011), the novel data presented in this manuscript may help explain in part the mechanisms that cause an increased association with NEC development in infants exposed to chorioamnionitis. Further experiments are ongoing that combine these two models to see whether FEMI influences induction of NEC-like injury.

There is an increasing understanding that compositional alterations of the microbiome are associated with development of NEC (Morrow et al., 2013; Elgin et al., 2016; Denning and Prince, 2018). It is then believed that these alterations impact TLR4 signal pathways to induce further inflammation, leading to injury to not only the intestine, but also the brain through the gut-brain axis (Moschopoulos et al., 2018; Nino et al., 2018; Niemarkt et al., 2019). Conventional dogma for over a century has been that the fetus develops within a sterile environment (Funkhouser and Bordenstein, 2013), although this has become controversial as several recent studies have detected a fetal microbiome using sterile-culture-independent techniques (Romano-Keeler et al., 2014; Martinez et al., 2018). While FEMI impacted components of the innate immune system such as Paneth cell and goblet cell biology, we did not find that it directly impacted the composition of the microbiome. Since FEMI occurs prior to birth and prior to or at the beginning of the development of the microbiome, it is reasonable to assume that environmental pressures would outweigh any direct effect FEMI may have on the microbiome (Ma et al., 2012). This is supported by the findings that the microbiome in preterm infants is assembled in a non-random pattern regardless of gestation (La Rosa et al., 2014). However, it is also possible that, although FEMI itself did not alter the microbiome composition, its impact on other mechanistic pathways may, in the presence of a secondary hit, then alter the microbial composition or the microbial metagenomics. This may be one reason that all animal models of NEC require multiple factors to initiate disease. These studies are of interest to the field and are currently areas of active investigation in our laboratory.

These deficits in normal intestinal defense induced by FEMI appear to be IL-6 driven. Acute chorioamnionitis is caused by either infection or cellular-stress-induced inflammation (Romero et al., 2014). Inflammation or infection of the placenta induces a robust signaling response, including increased concentrations of IL-1β, IL-6, IL-8 and TNF (Yoon et al., 1997; Mittal et al., 2008; Kim et al., 2015; Romero et al., 2015). Of these cytokines, IL-6 is significant as it effectively predicts pregnancy outcomes, and is critical to the development of end-organ injury induced by FEMI (Gomez et al., 1998; Blank et al., 2008; Hofer et al., 2013). Neonates diagnosed with fetal inflammatory response syndrome have an increased risk of developing severe neonatal morbidity, and this positively correlates with fetal plasma and amniotic fluid IL-6 concentrations (Gomez et al., 1998; Kim et al., 2001; Blank et al., 2008; Hofer et al., 2013). It is also well established that FEMI leads to abnormal neurodevelopment of the offspring (Boksa, 2010; Knuesel et al., 2014) by inducing region-specific changes in brain cytokine levels (Garay et al., 2013), and altering both the regulation and production of cytokines by T cells (Hsiao et al., 2012). Blockade of the IL-6 signaling pathways has been shown to prevent behavioral abnormalities that are typically seen in mice who have been exposed to maternal inflammation as fetuses (Smith et al., 2007). In our data, mice lacking the ability to produce IL-6 were protected from FEMI-induced intestinal injury and goblet cell loss. IL-6 is a potent cytokine that acts in a complex signaling pathway to modulate almost every aspect of the innate immune system (Liu et al., 1997; Hunter and Jones, 2015), which would help to explain the wide range of effects seen following exposure to FEMI. However, further studies need to be done to elucidate the source of IL-6 in older animals as adipose tissue has pro-inflammatory effects (Eder et al., 2009; Krüger et al., 2016) and the weight gain seen in older FEMI-exposed animals may contribute to serum IL-6 levels.

Our data looking at secondary exposure to LPS-induced inflammation also suggest the complexity of chorioamnionitis-associated disease processes. While NI exposure alone induced some of the highest levels of serum inflammatory markers, it was only when in combination with fetal inflammatory exposure that actual tissue injury was induced (Fig. 6). These effects may well be developmental-stage dependent. We have previously shown that neonatal exposure to TNF-induced inflammation can cause loss of mucin-positive goblet cells in 1-week-old mice while not invoking a compensatory increase in *Muc2* levels (McElroy et al., 2011). This is different from mice just a week older who experience both a significant loss of goblet cells and a significant increase in *Muc2* expression following the same TNF exposure (McElroy et al., 2011). In our data, NI given by LPS exposure at P5 induces significant increases in serum inflammatory markers, but does not induce intestinal injury or loss of goblet cells. Fetal exposure (FEMI) at E15.5, however, and more importantly the combination of both FEMI and NI (DI), induce both loss of goblet cells and significant intestinal injury patterns. While FEMI did not induce significant alterations in the composition of the microbiome, these inflammatory episodes are occurring during the fetal/neonatal timeframe and may represent changes in immune priming (Torow and Hornef, 2017). This is currently a focus of continued work in our laboratory.

In our study, we studied only male animals at the older ages to prevent any bias, as male animals are significantly heavier than females. Of note, we saw no other significant sex-driven differences in our model. There is a known sex-dependent difference in neonatal outcomes, with male infants having higher rates of morbidity and mortality than females, especially in neurocognitive outcomes (Stevenson et al., 2000). In human studies of male/female twins exposed to chorioamnionitis, the males had significantly higher amounts of placental inflammation and placental lesions when compared to their female siblings (Jahanfar and Lim, 2018). Rat studies using group B *Streptococci*-induced chorioamnionitis found an increase in placental inflammatory markers in male offspring compared to females (Allard et al., 2019), and sheep studies found that male lambs exposed to LPS-induced chorioamnionitis had lower lung volumes than females (Lambermont et al., 2012). However, it is important to note that none of these studies looked at long-term outcomes of the offspring or at GI physiology, which our novel data present. Nevertheless, it is possible that sex differences may play an as-yet-undefined role in gut pathology and should be studied further in the future.

Our data may have great relevance to the premature population. Infants exposed to intrauterine infection, depending on their degree of prematurity, will spend time in the neonatal intensive care unit (ICU), where they will be exposed to pathogens, indwelling catheters, augmented feeds and other foreign antigens, which can induce an inflammatory response. Based on our data, it is reasonable to project that infants who had FEMI and then are exposed to a subsequent inflammatory episode would experience an exaggerated drop in intestinal defenses and an exaggerated intestinal injury. This could well help explain the association between fetal exposure to chorioamnionitis and a subsequent increase in development of NEC (Been et al., 2013; García-Muñoz Rodrigo et al., 2014).

## MATERIALS AND METHODS

### Mice

All animal experiments were performed according to protocols approved by the University of Iowa Institutional Animal Care and Usage Committees. All experiments were performed using C57BL/6J mice or transgenic mice on a C57BL/6J background whose founders were purchased from Jackson Laboratories and who were housed under standard conditions in an Association for Assessment and Accreditation of Laboratory Animal Care (AAALAC)-approved vivarium. On day 15.5 of gestation (where copulatory plug=day 0), pregnant mice were given a single intraperitoneal injection of LPS (100 μg/kg body weight) derived from *E. coli O55:B5* (Sigma-Aldrich), or an equivalent volume of saline for sham controls (Fricke et al., 2018). Following injection, mice were returned to standard housing and pregnancy was allowed to continue uninterrupted until normal vaginal delivery. Approximately 50% of litters were live-born at term as previously described (Fricke et al., 2018), and these pups were subsequently raised under normal animal care conditions, weighed weekly, and then euthanized at P0, P7, P14, P28 or P56. To prevent size-based biases, only male mice were used for statistics at P28 and P56. Male and female numbers were roughly equal for P0-P14. Experiments evaluating the effects of a secondary exposure to inflammation were performed by injecting pregnant dams with LPS or saline as above. Newborn offspring were carried to term, delivered vaginally and raised under normal vivarium conditions. On P5, newborns were randomized to receive an intraperitoneal injection of LPS (100 µg/kg body weight) or an equivalent volume of saline. This led to four treatment groups: sham control, exposure to FEMI alone, exposure to NI alone, or exposure to both FEMI and NI. Following the secondary injection, pups were returned to their mother for 48 h and then euthanized.

### Histology

Ileal samples were defined as the distal 1/3 of the intestine between the stomach and the cecum. Tissue was collected and fixed in neutral buffered 10% formalin, embedded in paraffin, and sectioned at 5 µm thickness. The samples were stained with either hemotoxin and eosin (H&E) (structure), Alcian Blue (goblet cell quantification), or Periodic acid Schiff (PAS) and Alcian Blue (Paneth cell quantification). Tissue was evaluated under a Nikon microscope by two independent, blinded investigators, and counts were compared for consistency and congruence. Goblet cells were quantified per 1000 epithelial cells; Paneth cells were quantified per 100 intestinal crypts. Over 300 villi/crypt units were evaluated in each animal. Villous length, villous area, submucosa depth and muscularis thickness were measured and quantified using ImageJ software as previously described (Zhang et al., 2012; White et al., 2017b). Injury scores were determined via a three-point intestinal injury scoring scale (0=normal, 1=mild, 2=severe) based on degree of villi vacuolization, mucosal ulceration, lamina propria damage and presence of hemorrhage within villi as previously described (Wynn et al., 2016).

### Serum collection

Prior to euthanasia, blood was obtained from the facial vein as previously described (White et al., 2017a). Whole blood samples were placed on ice for 1 h then centrifuged at 7000 rpm (3287 ***g***) for 5 min to isolate serum. Cytokines were quantified using a Meso Scale Discovery V-Plex assay (Meso Scale, Gaithersburg, MD, USA) according to the manufacturer's instructions. Plates were read on a Sector Imager 2400 at 620 nm.

### qRT-PCR

Quantitative real-time reverse transcription-polymerase chain reaction (qRT-PCR) was performed using Taqman Fast Universal PCR Master Mix (2×) (Life Technologies) and Taqman Gene Expression Assays for *Muc2*, *Defa1*, cleaved caspase 3 and *Ki67* (Life Technologies). qRT-PCR reactions were run in a C1000 Thermal Cycler (Eppendorf) and using the CFX96 Real-Time PCR Detection System (Bio-Rad). Fold change in gene expression was determined by normalizing gene expression to β-actin in each sample. The 2ΔΔ-CT method was used to compare gene expression levels between samples. Serum from three or four pups in each of the second-hit groups was used.

### Microbiome analysis

Cecal microbial analysis was performed as previously described (Fricke et al., 2018; Lueschow et al., 2018). In brief, ceca were removed and placed in 1 ml of RNALater (Sigma-Aldrich, St Louis, MO, USA) and stored overnight at −4°C. The ceca were then transferred to a clean tube and stored at −80°C until processing. The ZR Fecal DNA MiniPrep kit (Zymo Research, Irvine, CA, USA) was used to extract DNA from the intact ceca, and extracted DNA was stored at −20°C. Amplification and sequencing were performed as previously described using the V4 domain and F515/R806 primers (Underwood et al., 2015; Fricke et al., 2018; Caporaso et al., 2011). PCR reactions used 5-100 ng DNA template, 1× GoTaq Green Master Mix (Promega, Madison, WI, USA), 1 mmol/l MgCl_2_ and 2 pmol of each primer. PCR was performed at 94°C for the initial 3 min followed by 35 cycles of 94°C for 45 s, 50°C for 60 s, and 72°C for 90 s, with a final extension of 72°C for 10 min. PCR amplicons were grouped at approximately equal amplification intensity ratios and were purified using the Qiaquick PCR purification kit (Qiagen). The PCR amplicons were submitted to the UC Davis Genome Center DNA Technologies Core for Illumina paired-end library preparation, cluster generation and 250 bp paired-end Illumina MiSeq sequencing. Data from the sequencing run was analyzed using the QIIME software package (University of Colorado, Boulder, CO, USA, version 1.9.1) (Caporaso et al., 2010b). Sequences were quality filtered and demultiplexed, and the UCLUST (drive5.com, Tiburon, CA, USA) was used to assign operational taxonomic units (OTUs) to the sequences, based on a 97% pairwise identity (Edgar, 2010; Bokulich et al., 2013). Secondary filtration of 0.005% was used to remove low-abundance OTUs (Bokulich et al., 2013). The filtered OTUs were taxonomically classified based on the Ribosomal Database Project classifier (Michigan State University, East Lansing, MI, USA) (Wang et al., 2007) against a representative subset of the Greengenes 16 s rRNA database (Second Genome, South San Francisco, CA, USA, gg_13_5 release) (DeSantis et al., 2006). OTU sequence alignment was performed using PyNAST (University of Colorado) (Caporaso et al., 2010a; Bokulich et al., 2013) and was used to construct a phylogenetic tree for β-diversity analyses. β-diversity was estimated by calculating unweighted and abundance-weighted UniFrac distances (Lozupone and Knight, 2005). Sample clustering was based on between-sample distances.

### Statistical analysis

All experiments were performed in at least triplicate and specific sample sizes are denoted in the results and/or figure legends. Non-parametric Kruskal–Wallis testing and Student *t*-tests were performed as appropriate to determine statistical significance using SAS v9.4 and Graph Pad Prism v8. Significance was set as *P*<0.05 for all experiments. In gene expression analyses, only results that were mathematically significant and greater than twofold different were considered clinically significant. All error bars are s.e.m. measurements.
